# Supplementary material for: Junction-based lamellipodia drive endothelial cell rearrangements in vivo via a VE-cadherin-F-actin based oscillatory cell-cell interaction
Source: Nat Commun. 2018 Aug 31;9:3545. doi: 10.1038/s41467-018-05851-9 (PMC6119192; doi:10.1038/s41467-018-05851-9)
Supplement: Supplementary file 1 — Supplementary Information [file 41467_2018_5851_MOESM1_ESM.pdf]

Supplementary Information

Paatero et al. "Junction-based lamellipodia drive endothelial cell rearrangements in vivo via a VE-cadherin-F-actin based oscillatory cell-cell interactions"

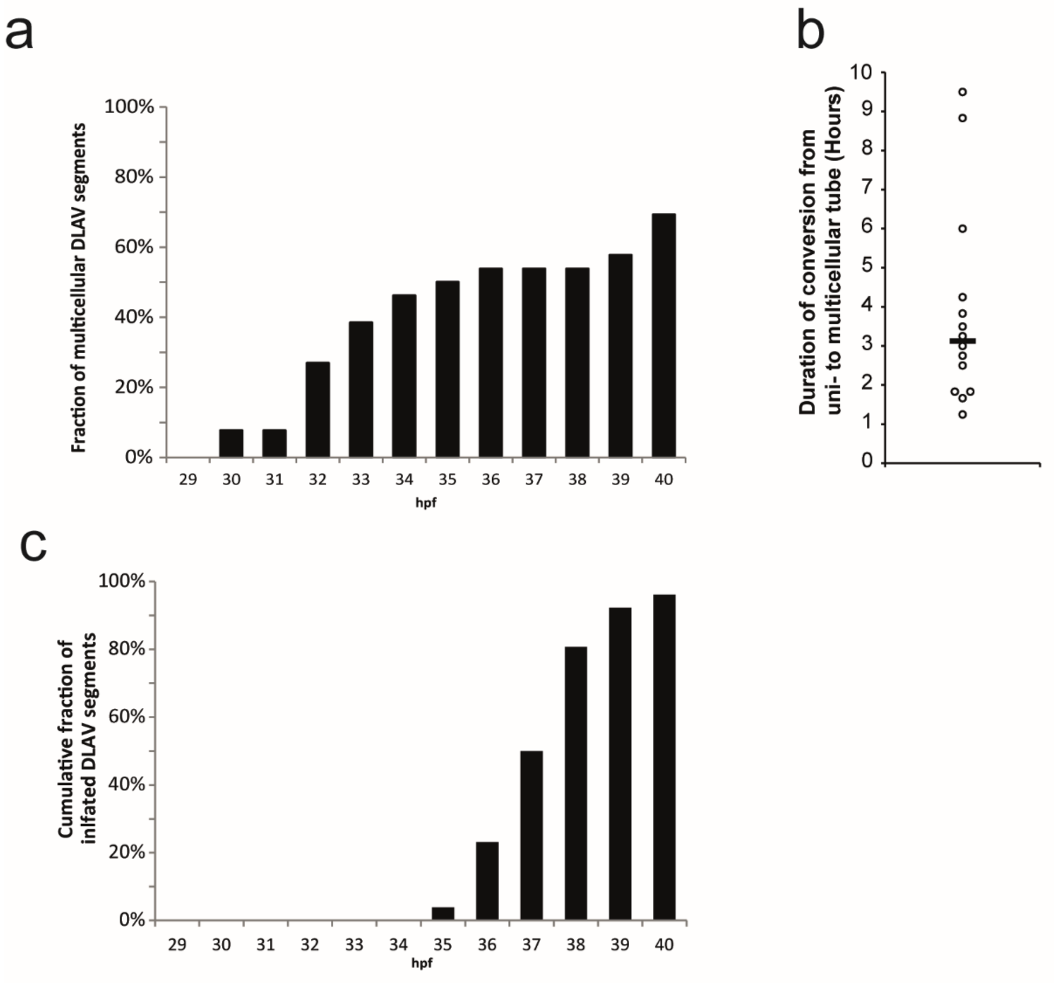

Supplementary Figure 1. *Description of the transition of dorsal longitudinal anastomosing vessel (DLAV) from unicellular to multicellular architecture.*

a) Quantification of the fraction of multicellular DLAV segments using Tg(BAC(*cdh5:cdh5-ts*)) embryos during development. n=26 DLAV segments (8 embryos). b) Quantification of the duration from anastomosis until final conversion into a multicellular tube. Black line is median. n=14 segments (8 embryos). c) Quantification of DLAV segments carrying blood flow during development. n=26 segments (8 embryos). hpf, hours post-fertilization.

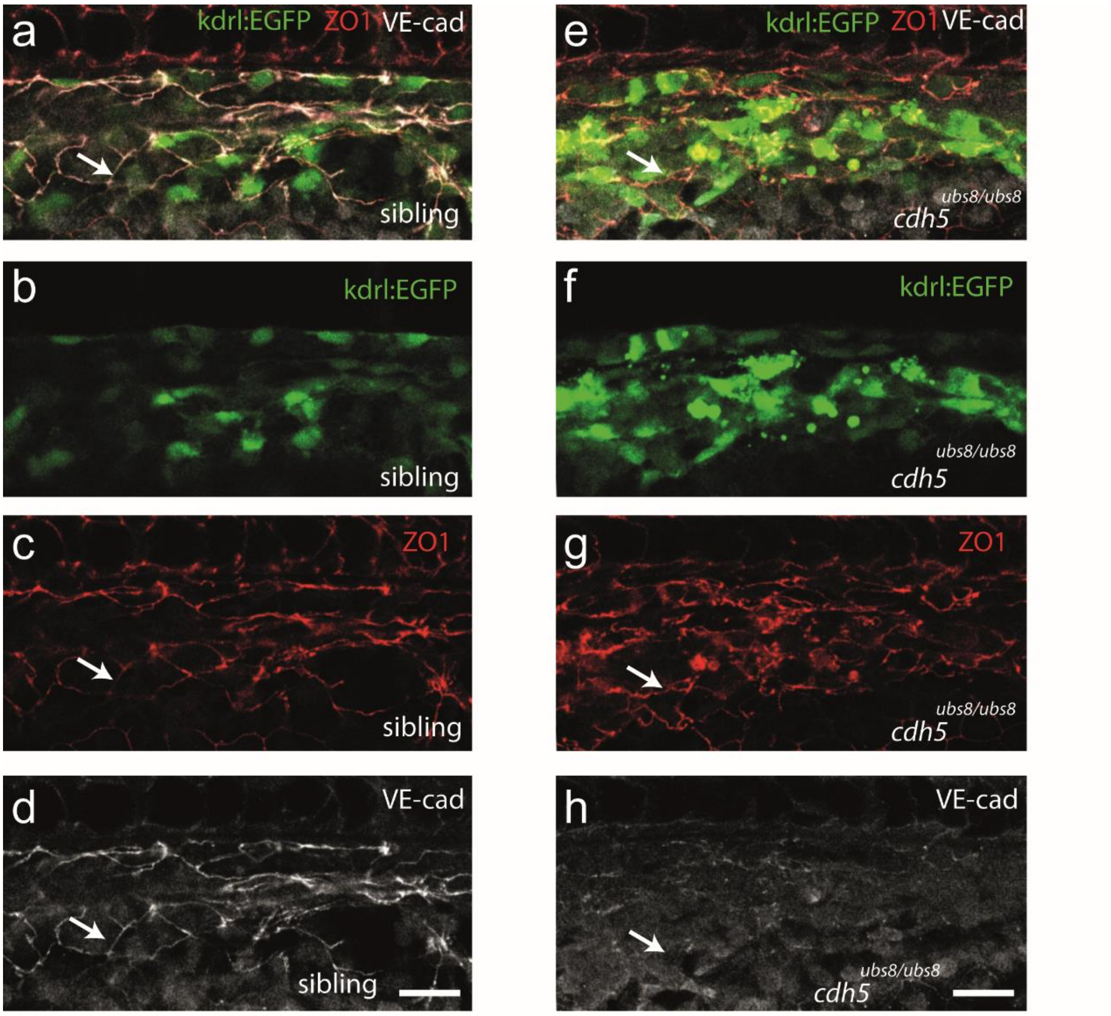

Supplementary Figure 2. *Validation of the rat Ve-cadherin antibody in zebrafish embryo whole-mount immunofluorescence analysis.*

a-h) Confocal images of a Tg(*kdrl:EGFP*^{s843}) wild-type sibling (a-d) or a VE-cad null mutant embryo (Tg(*kdrl:EGFP*^{s843});*cdh5*$^{ubs8/ubs8}$) (e-h), stained for VE-cadherin (rat anti-Cdh5) and ZO1. a and e shows the merged channels, b-d and e-h the individual channels. Arrow points to an endothelial cell-cell junction. Scale bar 20 µm.

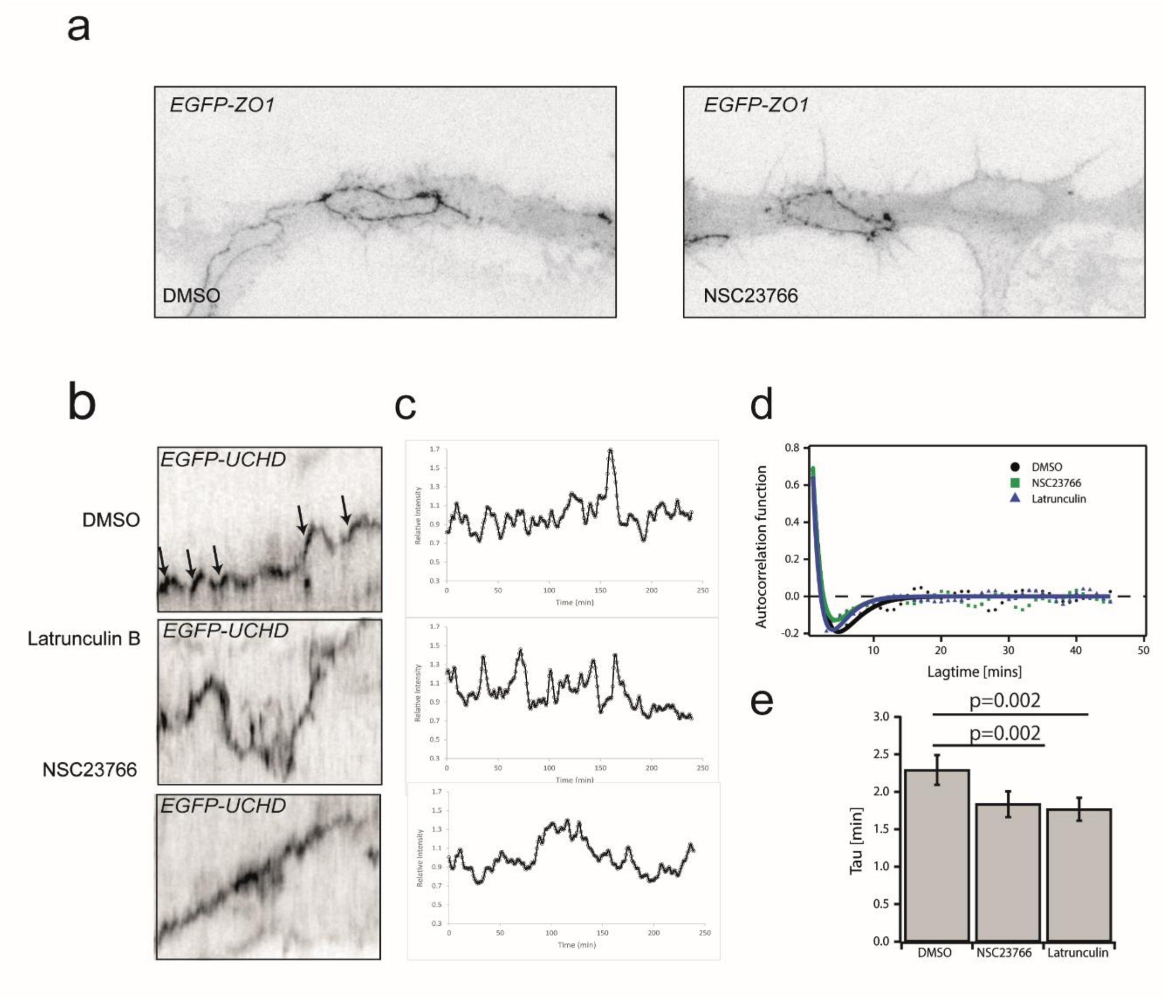

Supplementary Figure 3. *Analysis of F-actin oscillations of the remodeling junctions.*

a) Kymograph across the junction in dorsal aorta in EGFP-UCHD fish (data generated as in Fig. 3). Solid arrow denotes forward movement. b) Intensity plotting of EGFP-UCHD kymographs. c) Averaged autocorrelation functions of EGFP-UCHD intensity. DMSO, n=14 kymographs; Latrunculin B, n=16 kymographs; NSC23766, n=16 kymographs. d) Comparison of autocorrelation lifetime (tau parameter). Means and standard deviations are plotted. ANOVA and Dunnet´s post-hoc test was used.

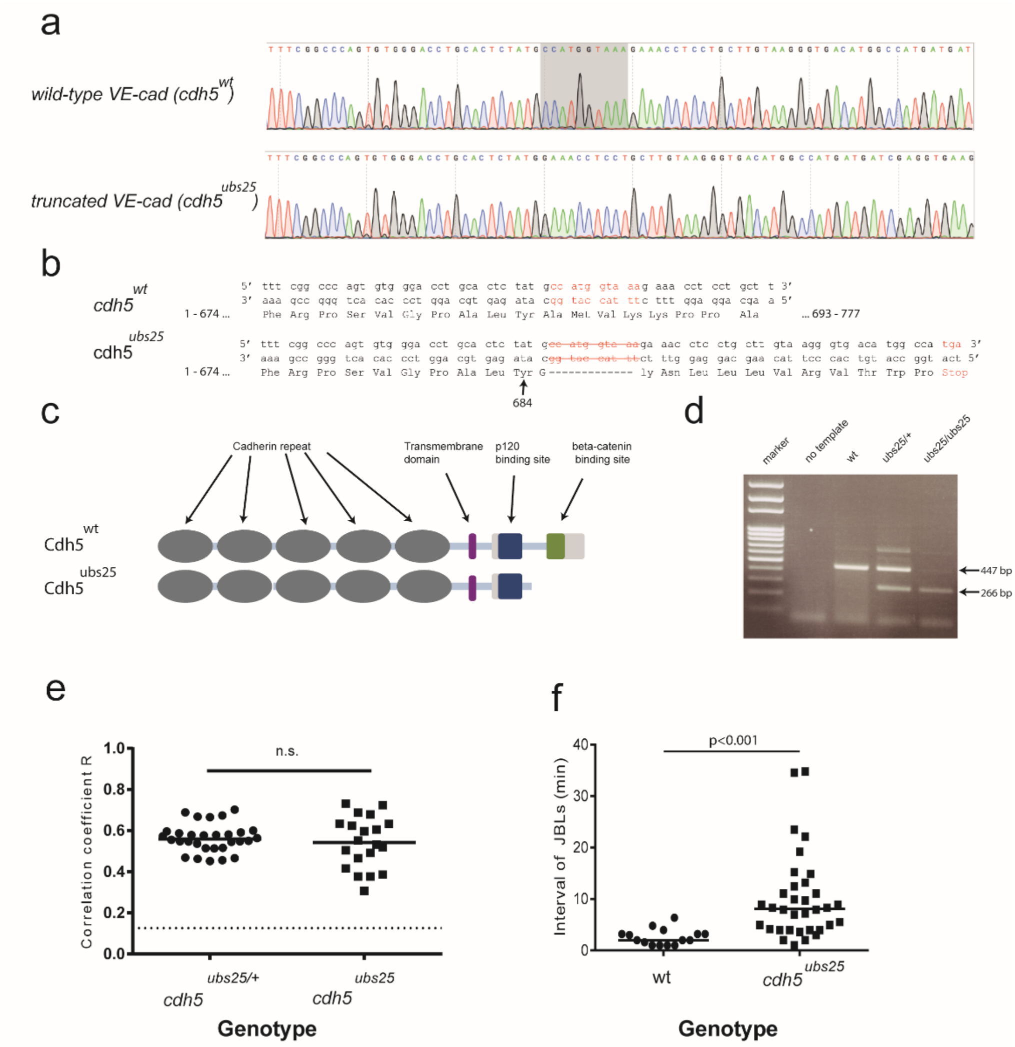

Supplementary Figure 4. *Generation and validation of VE-cad truncation mutant allele.*

a) Sequencing chromatogram of wild-type *ve-cadherin* (encoded by *cdh5* gene*)* sequence (exon 12) and respective truncation mutant *cdh5*^{ubs25} sequence. b) The wild-type and mutant DNA sequences and their respective translations. The *cdh5*^{ubs25} mutation leads to a premature stop. c) Schematic illustration of the domains in both full-length wild-type VE-cad and in truncated VE-cad (Cdh5^{ubs25}). d) Example of genotyping PCR and different *cdh5*^{ubs25} allelic combinations. e) Plot showing colocalization of ZO1 and truncated Cdh5 at endothelial cell junctions. Pearson correlation coefficients of colocalization analysis of anti-VEC and anti-ZO1 in heterozygote (*cdh5*$^{ubs25+/-}$) and homozygote (*cdh5*$^{ubs25/ubs25}$) VE-cad mutants. *cdh5*$^{ubs25/+}$, n=30 cell-cell junctions (2 embryos) ; *cdh5*$^{ubs25/ubs25}$, n=20 cell-cell junctions (1 embryo).n.s., non-significant (p=0.75, non-parametric Mann-Whitney test). f) Quantitation of the interval of subsequently occuring JBLs based on EGFP-UCHD signal. Time from end of the first JBL to emergence of new JBL in the same spot was measured ; wild-type n=15 (2 embryos) and *cdh5*$^{ubs25/ubs25}$ n=36 (4 embryos). All embryos carried EGFP-UCHD transgene Tg*(fli:GFF ; UAS:EGFP-UCHD).* Non-parametric Mann-Whitney statistical test was used.

a

56 hpf	no blood flow	blood flow only in DA and PCV/CV	normal blood flow
WT (n=32)	0%	0%	100% (32/32)
ubs25-het (n=37)	0%	0%	100% (37/37)
ubs25-mut (n=18)	61% (11/18)	39% (7/18)	0%
ubs8-het (n=39)	0%	0%	100% (39/39)
ubs8-mut (n=18)	100% (18/18)	0%	0%

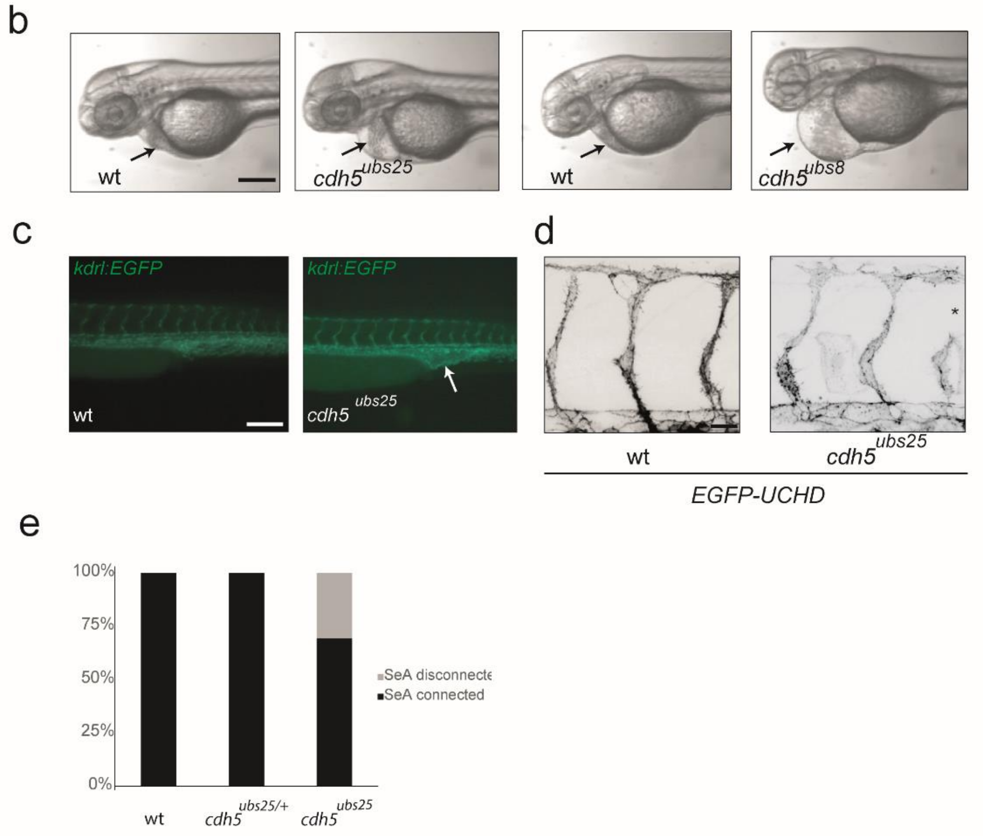

Supplementary Figure 5. *Phenotypic analysis of the ve-cad truncation mutant zebrafish.*

a) Table of blood flow phenotype of 56hpf VE-cadherin null mutant (*cdh5*$^{ubs8/ubs8}$) and the truncation mutant (*cdh5*$^{ubs8/ubs8}$) embryos. b) Stereomicroscope images of the embryos. Arrows point to pericardiac area, pronounced oedema is observed in mutant embryos. Scale bar, 400µm. c) Fluorescence images of Tg(*kdrl:EGFP*^{s843}) embryos at 48hpf. Arrow points of enlarged cardinal vein in *cdh5*^{ubs25} homozygote embryo. Scale bar, 200 µm. d) Confocal images illustrating the tip cell /stalk cell disconnection (arrow) in the *cdh5*^{ubs25} homozygote embryo. In normal SeA the connection between tip cell and stalk cell is intact. Scale bar, 20 µm. e) Quantification of tip cell stalk cell disconnection; *cdh5*$^{ubs25/+}$, n=7 SeA; *cdh5*$^{ubs25/ubs25}$, n=36 SeA; wild-type n=22 SeA.
