# Supplementary material for: Junction-based lamellipodia drive endothelial cell rearrangements in vivo via a VE-cadherin-F-actin based oscillatory cell-cell interaction
Source: Nat Commun. 2018 Aug 31;9:3545. doi: 10.1038/s41467-018-05851-9 (PMC6119192; doi:10.1038/s41467-018-05851-9)
Supplement: Supplementary file 3 — Description of Additional Supplementary Files [file 41467_2018_5851_MOESM3_ESM.pdf]

Description of Additional Supplementary Files

File Name: Supplementary Movie 1
Description: Time-lapse imaging of *VE-cad-Venus* (Tg(*BAC*(*cdh5:cdh5-ts*))) during DLAV formation starting at 29 hpf. Arrows point to edges of the elongating junction. Disappearance of junctions indicated that segment has achieved multicellular architecture. Time-lapse was imaged at 10min/stack (displayed at 5 fps).

File Name: Supplementary Movie 2
Description: High-resolution time-lapse imaging of *VE-cad-Venus* (Tg(*BAC*(*cdh5:cdh5-ts*)) in the junction of the DLAV around 32hpf. Some of the JBL are marked with an arrow.
Time-lapse was imaged at 60 sec/stack (displayed at 5 fps).

File Name: Supplementary Movie 3
Description: High-resolution time-lapse imaging of EGFP-UCHD (*Tg(fli:Gal4ff^{ubs3}; UAS:EGFP-UCHD^{ubs18}*)) in the junction of DLAV around 32 hpf. Some of the JBL are marked with an arrow. Time-lapse was imaged at 90 sec/stack (displayed at 5 fps).

File Name: Supplementary Movie 4
Description: High-resolution time-lapse imaging of EGFP-UCHD (*Tg(fli:Gal4ff^{ubs3}; UAS:EGFP-UCHD^{ubs18}*)) in the junction of DA around 32hpf. Some of the JBL are marked with an arrow. Time-lapse was imaged at 1min/stack (displayed at 10 fps).

File Name: Supplementary Movie 5
Description: High-resolution time-lapse imaging of mRuby-UCHD (red) and VE-cad-Venus (green) (*Tg(fli:Gal4ff^{ubs3};UAS:mRuby2-UCHD^{ubs20};BAC*(*cdh5:cdh5-ts*)) in the junction of DLAV around 32hpf. The time-lapse was imaged at 30 sec/stack (displayed at 5fps).

File Name: Supplementary Movie 6
Description: High-resolution time-lapse imaging of mRuby-UCHD (red) and EGFP-ZO1 (green) (*Tg*(*fli:Gal4ff^{ubs3};UAS:mRuby2-UCHD^{ubs20};UAS:EGFP-hZO1^{ubs5}*)) in the junction of DLAV around 32 hpf. The double-junctions are marked with arrows. Time-lapse was imaged at 12 sec/stack (displayed at 5 fps). Scale bar 1 µm.

File Name: Supplementary Movie 7
Description: High-resolution time-lapse imaging of mCherry-ZO1 (red) and VE-cad-Venus (green) (*Tg(BAC*(*cdh5:cdh5-ts*)*;fli:Gal4ff*^{ubs3}*;UAS:mCherry-ZO1*)) in the junction of SeA around 32 hpf. Time-lapse was imaged at 1 min/stack (displayed at 5 fps). Scale bar 1 µm.

File Name: Supplementary Movie 8
Description: High-resolution time-lapse imaging of DMSO-treated embryo expressing *VE-cad-Venus* (Tg(*BAC*(*cdh5:cdh5-ts*))) in the junction of DLAV around 32hpf. Time-lapse was imaged at 60 sec/stack (displayed at 5 fps).

File Name: Supplementary Movie 9
Description: High-resolution time-lapse imaging of Latrunculin B-treated embryo expressing *VE-cad-Venus* (Tg(*BAC*(*cdh5:cdh5-ts*))) in the junction of DLAV around 32hpf. Time-lapse was imaged at 60 sec/stack (displayed at 5 fps).

File Name: Supplementary Movie 10
Description: High-resolution time-lapse imaging of NSC23766-treated embryo expressing *VE-cad-Venus* (Tg(*BAC*(*cdh5:cdh5-ts*))) in the junction of DLAV around 32hpf. Time-lapse was imaged at 60 sec/stack (displayed at 5 fps).

File Name: Supplementary Movie 11
Description: Video of blood flow in the trunk of 2dpf wild-type and cdh5(ubs25) mutant embryos (both mild and severe phenotypes are shown).
